# A Case of Primary Malignant Melanoma of the Nasolacrimal Duct

**DOI:** 10.7759/cureus.98432

**Published:** 2025-12-04

**Authors:** Kosuke Terazawa, Ryo Utakata, Hisakazu Kato, Munekazu Matsumoto, Takenori Ogawa

**Affiliations:** 1 Department of Otolaryngology - Head and Neck Surgery, Gifu University Graduate School of Medicine, Gifu, JPN; 2 Department of Plastic and Reconstructive Surgery, Gifu University Hospital, Gifu, JPN; 3 Department of Pathology, Gifu University Hospital, Gifu, JPN; 4 Cancer Center, Gifu University Hospital, Gifu, JPN

**Keywords:** immune checkpoint inhibitors, mucosal malignant melanoma, nasolacrimal duct, postoperative radiotherapy, surgery

## Abstract

Malignant melanoma of the nasal cavity and paranasal sinuses is an extremely rare disease, with only a few reported cases originating from or extending into the nasolacrimal duct. Multimodal treatments, including surgery, radiotherapy, chemotherapy, immunotherapy, and immune checkpoint inhibitors, have been attempted; however, the treatment outcomes remain unsatisfactory. We herein report a case of primary malignant melanoma of the nasolacrimal duct, which was successfully managed through complete en bloc resection of the nasolacrimal duct along with the surrounding bony structures, lacrimal sac, and puncta, followed by adjuvant radiotherapy and administration of immune checkpoint inhibitors. This multidisciplinary approach resulted in a favorable clinical course. Due to the rarity of mucosal malignant melanoma, many aspects of the disease remain unclear, and further evidence is warranted.

## Introduction

Malignant melanoma accounts for less than 5% of all malignant tumors of the nasal cavity and paranasal sinuses, making it a rare entity [1]. Among these, cases originating from or extending into the nasolacrimal duct are exceedingly uncommon. Multimodal treatment strategies, including surgery, radiotherapy, chemotherapy, immunotherapy, and immune checkpoint inhibitors, have been attempted; however, treatment outcomes remain suboptimal [2].

Here, we report a rare case of primary malignant melanoma of the nasolacrimal duct that was successfully managed by en bloc resection of the nasolacrimal duct, including the surrounding bony structures, lacrimal sac, and puncta, resulting in a favorable clinical course.

## Case presentation

**Figure 1:**
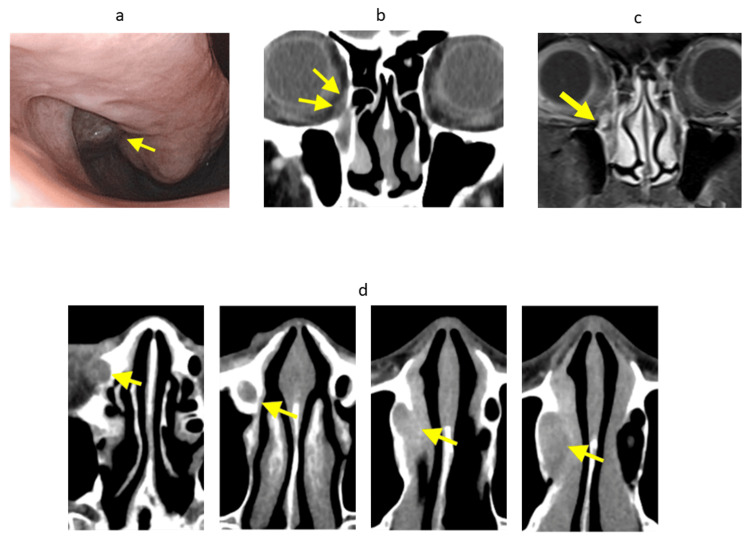
Preoperative endoscopic, CT, and MRI findings showing tumor extension from the inferior nasal meatus to the lacrimal sac (a) nasal endoscope, (b) coronal CT scan, (c) coronal MRI, and (d) axial CT scan. The tumor extended from the inferior nasal meatus along the nasolacrimal duct, reaching the lacrimal sac. Arrow: tumor, CT: computed tomography, MRI: magnetic resonance imaging

A 70-year-old woman presented with epistaxis as her chief complaint. She had no significant past medical history. Upon visiting a local clinic, a nasal mass was identified, and she was referred to our department for further evaluation. Initial examination revealed a tumor approximately 1 cm in diameter located in the right inferior nasal meatus (Figure 1a). A biopsy led to the diagnosis of malignant melanoma. The tumor was situated near the opening of the nasolacrimal duct, and contrast-enhanced CT and MRI showed partial enhancement within the right nasolacrimal duct (Figure 1b-1c). Further extension was observed toward the inferior wall of the orbit, suggesting tumor spread along the nasolacrimal duct. PET-CT demonstrated FDG uptake consistent with the tumor, but there was no evidence of regional lymph node or distant metastases. Based on these findings, the lesion was clinically diagnosed as nasal malignant melanoma, cT4aN0M0. As a result of the multidisciplinary team discussion involving otolaryngology-head and neck surgery, plastic surgery, ophthalmology, radiology, and oncology, upfront surgery was performed. Given the lack of evidence supporting neoadjuvant therapy for nasolacrimal duct malignant melanoma and the feasibility of complete resection, surgery was considered the most appropriate initial treatment modality.

Surgical findings

**Figure 2:**
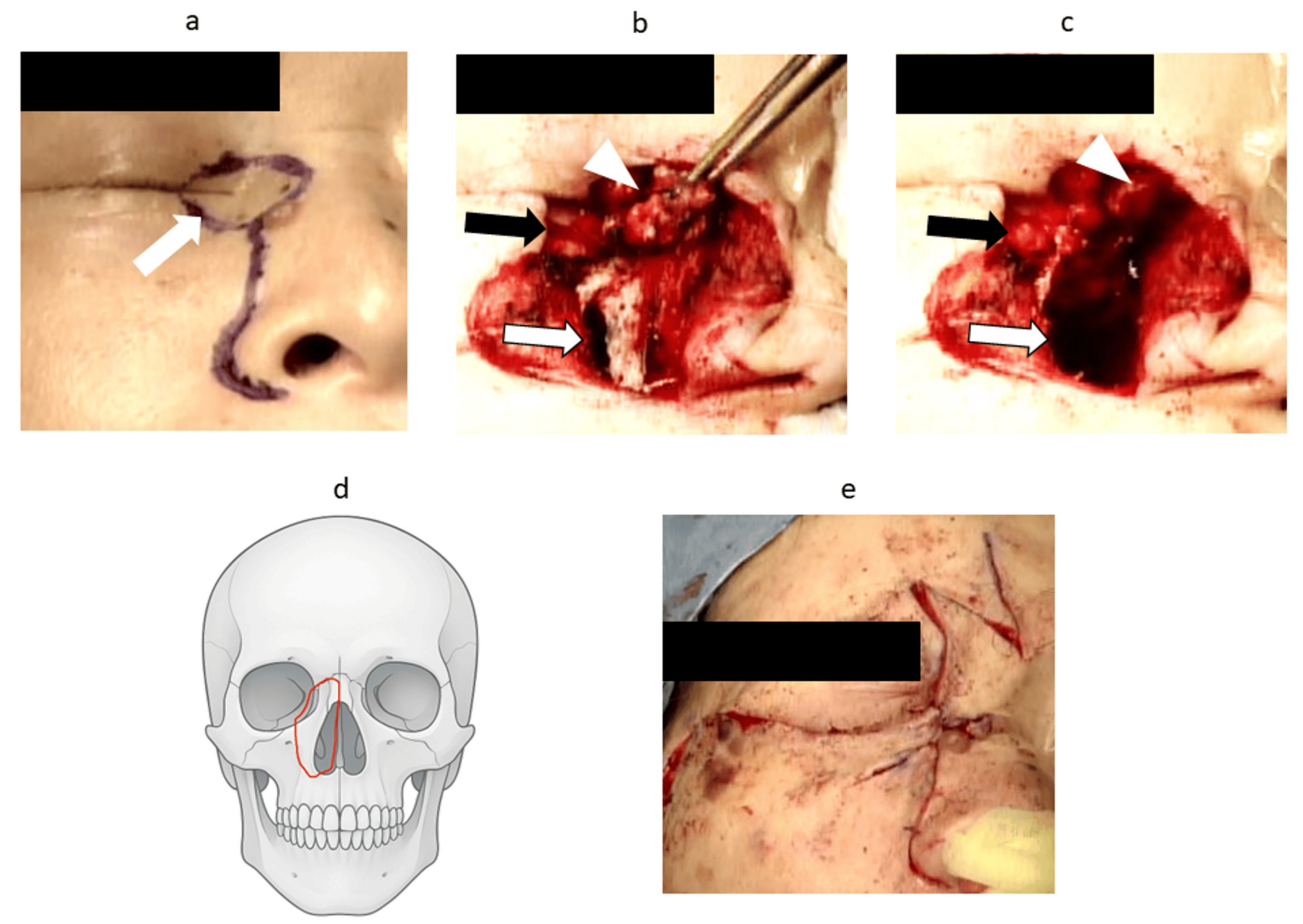
Intraoperative views demonstrating en bloc resection of the tumor via right lateral rhinotomy and subsequent reconstructive procedures (a) Skin incision (arrow) showing en bloc resection including the lacrimal puncta and overlying skin. (b) Tumor and resection margins (photograph). (c) Post-resection view; the black arrow indicates exposed fat after excision of the medial canthal conjunctiva, the white arrow indicates the maxillary bone wall resection line, and the white arrowhead indicates the tumor. (d) Schematic of the bone resection line; the red line indicates the planned surgical margins, including the lacrimal sac, medial orbital wall, and frontal process of the maxilla. The medial canthal conjunctival defect was not repaired, as it did not cause ocular displacement (Image Credit: Authors, using Microsoft PowerPoint and Paint 3D for Windows). (e) Reconstruction using glabellar and malar flaps after tumor and bone resection. Right lateral rhinotomy was performed with en bloc resection of the skin, lacrimal puncta, tumor, and surrounding bony structures, followed by reconstruction with glabellar and malar flaps.

A right lateral rhinotomy was performed with en bloc resection of the overlying skin, including the lacrimal puncta (Figure 2a). The anterior wall of the maxillary sinus was opened, and the bony floor was drilled down to the base of the maxillary sinus. The nasal bone was removed on the medial side of the nasolacrimal duct. Superiorly, the frontal process of the maxilla and inferiorly, the medial wall of the maxillary sinus were resected. The medial orbital wall was dissected free from the orbit, and the posterior end of the inferior turbinate was transected. The tumor was resected en bloc (Figure 2b-2d). Reconstruction was performed using glabellar and malar flaps (Figure 2e). Intraoperative frozen section and final pathological examination confirmed negative surgical margins.

Histopathological findings

**Figure 3:**
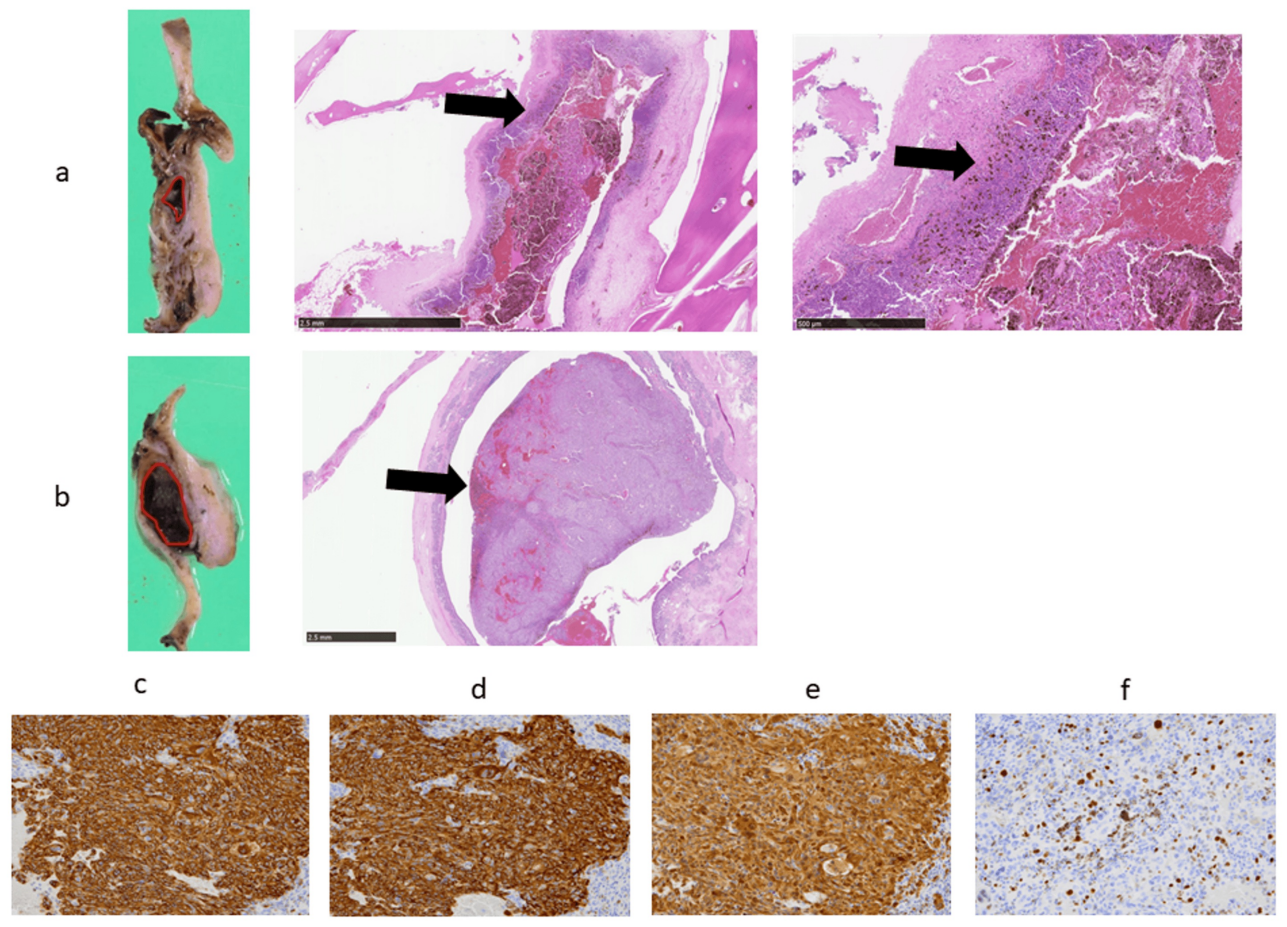
Histopathological and immunohistochemical findings confirming primary malignant melanoma of the nasolacrimal duct (a) Nasolacrimal duct level – gross appearance (left), H&E staining (×2, middle), and H&E staining (×20, right). (b) Inferior nasal meatus level – gross appearance (left) and H&E staining (×2, middle); red line indicates tumor (gross), black arrow indicates tumor (histology). A black, polypoid lesion protruding into the nasal cavity was observed. H&E staining showed atypical spindle- to polygonal-shaped cells with eosinophilic cytoplasm and prominent nucleoli located beneath the ciliated columnar epithelium. The tumor was also present beneath the nasolacrimal duct mucosa, whereas the inferior nasal meatus was spared, suggesting primary origin from the nasolacrimal duct. (c-f) Immunohistochemical staining for HMB-45 (c), Melan A (d), S100P (e), and MIB-1 (f). Tumor cells were positive for HMB-45, Melan A, and S100P, with an MIB-1 labeling index of approximately 30–40%. H&E: hematoxylin and eosin

A black, polypoid lesion measuring 25 × 8 × 6 mm was observed protruding from the nasolacrimal duct into the nasal cavity (Figure 3). Histopathological examination revealed that the surface was covered with ciliated columnar epithelium, and the main tumor component was located in the stroma. The lesion consisted of medium- to large-sized atypical cells with round-to-spindle-shaped morphology, prominent nucleoli, distinct nuclear membranes, and eosinophilic cytoplasm, exhibiting diffuse infiltrative growth. Brown pigment deposits, consistent with melanin, were observed in the cytoplasm of some tumor cells, and multinucleated atypical cells were also noted. The tumor was also present beneath the nasolacrimal duct mucosa, whereas the inferior nasal meatus was spared, suggesting primary origin from the nasolacrimal duct. (Figure 3a-3b). Mitotic figures were observed at approximately 5 per 10 high-power fields. Immunohistochemically, the tumor cells were positive for S100P, HMB-45, and Melan A, but negative for BRAF V600E. The MIB-1 labeling index was 30-40% (Figure 3c-3f). These findings were consistent with malignant melanoma. Tumor invasion was generally confined to the mucosa, without evidence of vascular invasion. The surgical margins were negative for tumor involvement. Mild medial eyelid closure impairment and epiphora were observed due to resection of the medial canthus. However, no visual functional deficits such as diplopia or decreased visual acuity were noted.

**Figure 4:**
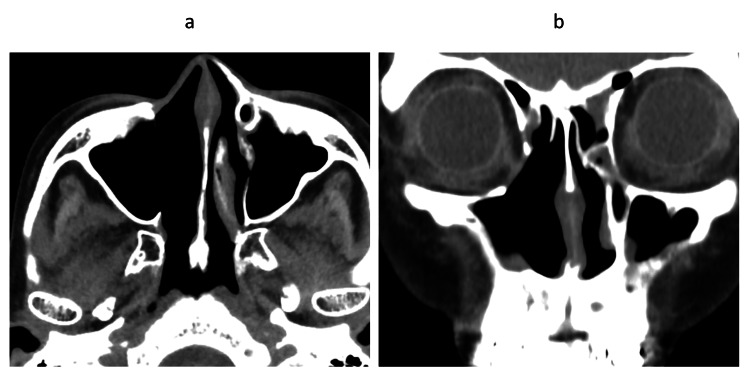
Postoperative follow-up CT images three years after surgery showing no evidence of recurrence (a) Axial view and (b) coronal view. No recurrence is observed in either image. CT: computed tomography

Postoperative treatment included radiotherapy (60 Gy in 30 fractions) followed by adjuvant immunotherapy with nivolumab. After two administrations, the patient developed thyrotoxicosis (FT3: 6.18 pg/mL (reference range: 2.3-4.0 pg/mL), FT4: 2.36 ng/dL (reference range: 0.9-1.7 ng/dL), TSH: 0.088 μIU/mL (reference range: 0.61-4.23 μIU/mL)), and treatment was discontinued due to immune-related adverse events. At three years postoperatively, the patient remains free of recurrence (Figure 4).

## Discussion

Approximately 10-25% of malignant melanomas occur in the head and neck region [3]. Among mucosal melanomas of the head and neck, the nasal cavity and paranasal sinuses are the most common primary sites, accounting for 59-80% of cases [4]. However, malignant melanoma of the nasal cavity and paranasal sinuses is rare, representing less than 5% of all malignant tumors in this region. Of these, 70-80% originate from the nasal septum [5].

Reports of nasal cavity malignant melanoma extending along the nasolacrimal duct are extremely rare. In our literature review, we identified only three cases of primary nasolacrimal duct melanoma and one case with orbital invasion via the nasolacrimal duct (Table 1) [6-8]. Even in these rare cases, multimodal treatment with lateral rhinotomy and postoperative radiotherapy was performed. However, early postoperative recurrence has been frequently reported.

**Table 1:** Reported cases of mucosal melanoma involving nasolacrimal duct OMC: osteomeatal complex, NLD: nasolacrimal duct, RT: radiotherapy

Author/year	Age/sex	Symptoms	Imaging	Treatment	Margin	Adjuvant therapy	Outcome
Lewis et al. 2006 [6]	41/M	Bloody tears, epistaxis	NLD mass → OMC/ethmoid	operation	Clear	RT + dendritic vaccine	No local recurrence at 3 years; bone metastases developed, died 6 months later
Esteban et al. 2007 [7] (case 1)	77/M	Bloody tears, epistaxis	NLD mass	operation	N/A	RT	Liver metastases at 6 months; died 3 months later
Esteban et al. 2007 [7] (case 2)	60/M	Nasal obstruction	NLD mass	operation	Clear	RT	No recurrence at 1 year
Chou et al. 2010 [8]	78/M	Epiphora, periorbital swelling	Nasal cavity → NLD → orbit	RT alone	N/A	Chemotherapy + Immunotherapy	Lung metastasis at 3 months; dead at 6 months
Present case	70/F	Epistaxis	Nasal cavity → NLD → orbit floor (T4a)	operation	Clear	RT + nivolumab	No recurrence at 3 years

Mucosal melanomas are known to have a poorer prognosis than cutaneous melanomas, although resectable mucosal melanomas should be surgically removed, as with cutaneous melanomas. Radical resection is generally recommended for malignant melanoma; however, achieving adequate surgical margins is often challenging in the head and neck due to the complex three-dimensional anatomy of mucosal structures.

Aside from melanoma, only a few cases of primary malignancies of the nasolacrimal duct have been reported. Some authors have demonstrated that en bloc resection of the nasolacrimal duct along with surrounding bony structures can achieve reasonable local control [9]. In our case, we performed tumor resection via lateral rhinotomy with en bloc resection of the skin, including the lacrimal puncta, thereby removing the nasolacrimal duct and lacrimal sac entirely. In addition, the nasal bone and frontal process of the maxilla were resected to achieve complete tumor removal.

Postoperative radiotherapy is reported to be effective for local control, although some studies suggest it does not significantly improve overall survival [10]. According to the National Comprehensive Cancer Network guidelines, radiotherapy is strongly recommended for Stage III disease and is recommended for Stage IVa, regardless of surgical margin status [11]. Additionally, particle beam therapy has shown efficacy for non-squamous cell carcinomas. One study of 33 cases of sinonasal malignant melanoma reported a two-year local control rate of 83% with particle therapy [12].

The efficacy of immune checkpoint inhibitors has been well established for cutaneous melanoma, and similar treatment strategies are increasingly applied to mucosal melanoma. For stage III and IV disease, adjuvant nivolumab therapy is beneficial [13] and was administered in the present case.

For unresectable cases, evaluation of PD-L1 expression and BRAF mutations is recommended. Based on these biomarkers, treatment options may include anti-PD-1 antibodies, anti-CTLA-4 antibodies (either as monotherapy or in combination), or combination therapy with BRAF and MEK inhibitors, or BRAF inhibitor monotherapy. Combination immunotherapy (anti-PD-1 plus anti-CTLA-4) has shown higher response rates than monotherapy [14], but it also carries a high risk of severe adverse events, affecting more than half of patients. Furthermore, evidence regarding efficacy and safety in previously treated patients remains limited [15].

Genetic mutations in mucosal melanoma are reportedly one-fifth to one-tenth as frequent as in cutaneous melanoma [16], and BRAF mutations are particularly rare in mucosal subtypes. Due to its rarity, many aspects of mucosal malignant melanoma remain unclear. Prospective observational studies, such as those currently being conducted by the Japan Clinical Oncology Group, are expected to contribute to the development of future evidence in this field.

## Conclusions

We experienced a rare case of malignant melanoma with invasion into the nasolacrimal duct. En bloc resection of the nasolacrimal duct, including the surrounding bony structures, lacrimal sac, and puncta, followed by postoperative radiotherapy and adjuvant immune checkpoint inhibitor therapy, resulted in a favorable clinical outcome. This case demonstrates that aggressive surgical resection with adjuvant immunotherapy may improve local control in select patients.
